# Digital model superimpositions: are different software algorithms equally accurate in quantifying linear tooth movements?

**DOI:** 10.1186/s12903-022-02129-x

**Published:** 2022-03-31

**Authors:** Samar M. Adel, Nikhilesh R. Vaid, Nadia El-Harouni, Hassan Kassem, Abbas R. Zaher

**Affiliations:** 1 Department of Orthodontics, Faculty of Dentistry, Alexandria University, Champollion Street, El Azarita, Alexandria, Egypt; 2 Department of Orthodontics, Saveetha Dental College, Saveetha Insitute of Medical and Technical Sciences, Chennai, India

**Keywords:** Registration, 3D digital models, 3D tooth movement, Digital orthodontics, Aligner therapy, Scanning, Digital Setup

## Abstract

**Background:**

To evaluate the accuracy of three different 3D digital model registration software packages for linear tooth movement measurements, with reference to a 3D digital virtual setup (DS).

**Methods:**

Twenty maxillary and mandibular pre-treatment scans of patients undergoing clear aligner therapy were used. Digital Setups were generated from pre-treatment scans using OrthoAnalyzer software. Both the pretreatment digital scans (T1) and Digital Setups (T2) were converted to STL files to be imported to the three studied software packages: Geomagic, OrthoAnalyzer and Compare. Linear changes in tooth positions were calculated for all the registered pairs.

**Results:**

The change in tooth position was compared between the calculated tooth movement using each of the registration software packages versus the actual generated tooth movement from the Digital Setups. Continuous data was expressed as mean and standard deviation. Intraclass Correlation Coefficients for agreements between Digital Simulation and each software was used. Intra and Inter-examiner reliabilities were also assessed using Intraclass Correlation Coefficients. Significance of the obtained results was expressed at p ≤ 0.01. Geomagic software showed agreements > 0.90 for maxillary linear tooth movements and between 0.75 and 0.90 for mandibular measurements. OrthoAnalyzer software showed agreements between 0.50 and < 0.75 for maxillary and mandibular measurements. Compare software showed agreements > 0.90 for maxillary and mandibular linear tooth movements, indicating the best consistency.

**Conclusions:**

Compare and Geomagic software packages consistently showed maximum accuracy in measuring the amount of tooth movement in the maxillary arch compared to the reference standard. Compare software showed the highest agreements in the mandibular arch. None of the three studied software packages showed poor agreement with the Digital Setup across all tooth movement measurements. Buccolingual tooth movements showed the highest agreements amongst linear measurements.

## Background

Appraisal of tooth movement, through digital superimpositions is critical in contemporary orthodontic protocols. Careful evaluation and quantification of superimpositions, enable professionals to understand capabilities and limitations of appliances and mechanics employed therein [1–5].

Digital intraoral models derived either indirectly from model scans or directly by intraoral scans are the first step in obtaining a detailed 3D representation of the dentition, on which planning, tracking, measurements, as well as simulations are performed [6–9]. Tooth movement can be quantified by registering serial 3D models acquired at different time points where they can be combined in the same spatial coordinate system [10]. Various techniques and software packages have been used for 3D digital registration of virtual models as well as for tooth movement measurements, so as to quantify treatment effects between time lines.

Software packages differ in the registration methods they offer, in the method of measuring 3D tooth movements, in costs, as well as in the time and complexity to perform a specific task [11, 12]. Accurate registration of serial models on stable and identifiable structures is mandatory [13].

Available software packages for model registration use a combination of computer-based algorithm and operator data-input. Software packages work by combining large amounts of data with fast, iterative processing and intelligent algorithms, allowing them to learn automatically from patterns or features in the data [14, 15]. Recently, registration software packages have been marketed which operate solely based on algorithms with minimal operator input [16].

Algorithms available for 3D registration include 1) Surface-based (registration based on surface area to bring the two preoperative and postoperative models to fit to each other) e.g. Geomagic software [17], 2) Landmark-based (software-assisted best-fit registration of arbitrary selected anatomical points/specified area) e.g. OrthoAnalyzer software [18], or 3) Information theory and mathematical algorithm technique-based (software-assisted superimposition of the registered structures) e.g. Compare software [19]. Landmarks and regions of interest used for registration should fulfill the basic requirement of being stable during growth and unchanged by bone modeling associated with orthodontic tooth movement especially during space closure after extractions [13]. Up till now, there is no consensus in the literature regarding the techniques to superimpose serial 3D intraoral digital models [3].

Several limitations exist in current literature comparing different registration techniques [3]. Most of them [20, 21] assessed the reliability of the superimposition technique in comparison to cephalometric radiographs which themselves are unreliable. Studies using implants as standard reference [22, 23] did not evaluate precisely the stability of the implants in the first place. In addition, most studies [11, 24] have compared their tooth movement outcomes with a doubtful self-assumed standard reference point/plane, which has not been validated. Studies have used different software packages that register digital models and assess 3D tooth movements to quantify treatment effects, but validation of the package itself remains untested. This study aims to answer this knowledge gap.

The aim of this study was to evaluate the accuracy of three different 3D digital model registration software packages that measure the amount of tooth movement, using predetermined amount of simulated tooth movement on a 3D Digital Setup, as a reference standard.

The null hypothesis was that there is no agreement between the predetermined tooth movement generated by the Digital Setup and the different digital model registration software packages in measuring the amount of linear tooth movements evaluated.

## Materials and methods

### Study design

This diagnostic accuracy and agreement study followed a modification of the Guidelines for Reporting Reliability and Agreement Studies (GRRAS) where each software package was considered as a rater [25]. IRB approval was obtained at the Faculty of Dentistry, Alexandria University (IRB: 00010556-IORG: 0008839), and informed consents sought from the subjects whose scans were used as a study material. Access to the original scans was limited to the principal investigator. All potentially identifiable patient information were removed from the scans. Scans were stored in one computer terminal connected to a protected server. The minimal sample size was calculated based on previous studies that evaluated the reliability of newly developed software calculating 3D tooth movements [12, 26]. Based on the results, a sample size of 20 scans was deemed enough to conduct this agreement study [27], with minimum accepted reliability ρ_0_ = 0.6 and maximum expected reliability ρ_1_ = 0.9, *k* = 3, where k corresponds to the number of tested software packages. The statistical significance alpha was set at 0.01 to account for multiple comparisons and a statistical power, 1 − β = 0.9. The minimum calculated sample size was 18, increased to 20 to account for defective scans.

### Sample collection

The sample consisted of full arch pretreatment maxillary and mandibular intraoral digital scans of actual adult patients undergoing CAT. All scans were randomly selected from the records of a single orthodontic office in Mumbai, India with more than 15 years of experience with CAT. A random number list of 20 was generated using Microsoft Excel from the total number of scans available in the office archive. The scanner used was a TRIOS 3-D intraoral scanner (3Shape, Copenhagen, Denmark). The scan data was then exported in STL format file extension and the files were imported into the three studied software packages and analyzed in the Department of Orthodontics, Alexandria University. The study group comprised scans of 20 patients with a Little’s irregularity index that ranged from 4 to 6 mm. All teeth in both arches were evaluated for 3D tooth movements except for third molars. The inclusion criteria for the scans were (1) Adult subjects treated with CAT who received treatment in both arches, (2) Scans had to be complete and of acceptable quality with a full complement of teeth except for the third permanent molars. Scans were excluded if (1) Treatment involved extraction of permanent teeth, (2) Teeth had surface anomalies or if (3) Scans had soft-tissue lesions covering the palate or the mucogingival junction (MGJ) of the mandibular arch, (4) Scans that had any previous history of orthodontic /orthopedic treatment.

**Fig. 1:**
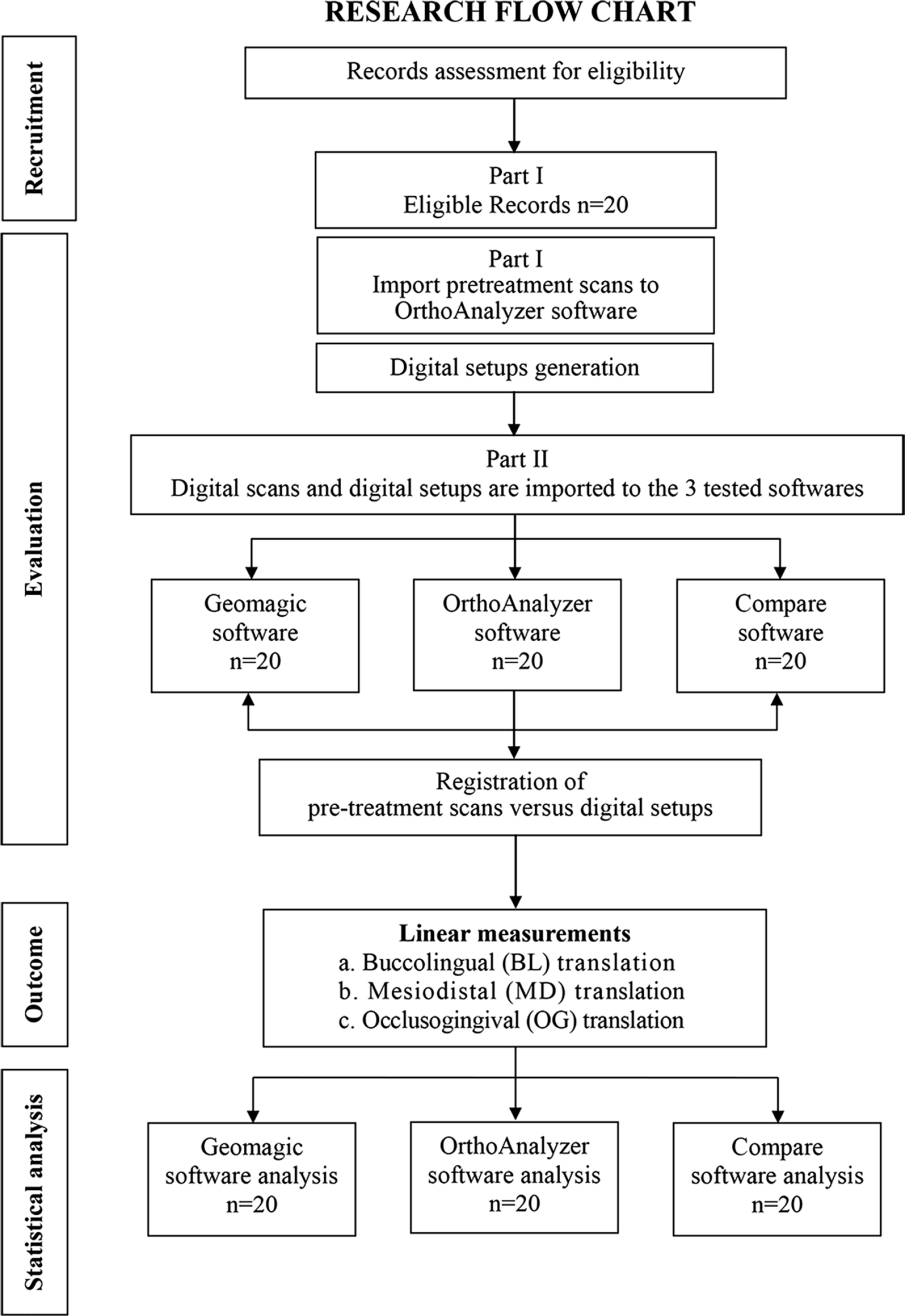
Research flowchart

All the scans that met the eligibility criteria were given an identification number. All digital scans were de-identified by an independent investigator, and imported into the three different tooth measuring software programs for the principal investigator to evaluate (Fig. 1).

### Procedure

#### Digital Setup

**Fig. 2:**
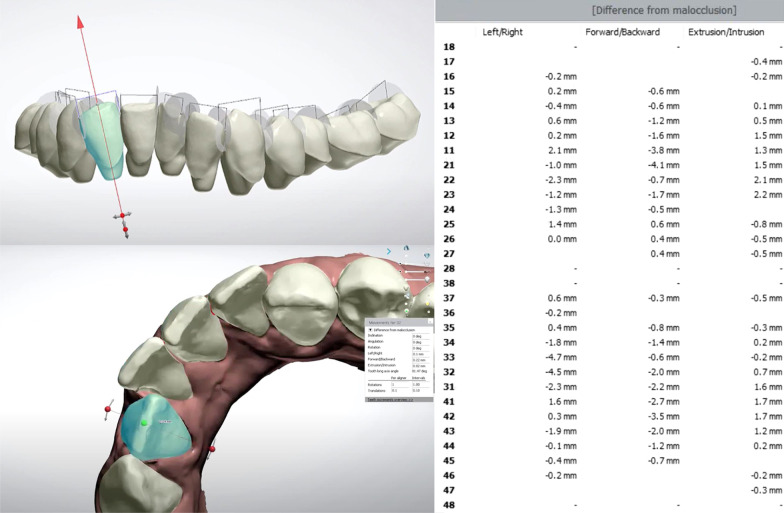
Segmentation of teeth and virtual tooth movements during Digital Setup generation

Full arch maxillary and mandibular pretreatment scans (T1) were imported to OrthoAnalyzer software (3Shape Ortho System, Copenhagen, Denmark). Scan preparations were deemed necessary for all maxillary and mandibular pretreatment scans. Models were trimmed and oriented into three planes. Virtual Digital Setups were done by using virtual segmentation techniques. After defining the mesial and distal edges of each tooth, the software automatically creates a cut spline for each tooth that can be manually adjusted if needed. The original occlusal plane and vertical planes were determined and used as a reference. The long axis of each tooth was determined and teeth were moved virtually to their desired ideal position in the arch. All linear tooth movements were visualized and quantified in three directions (buccolingual BL, mesiodistal MD, occluso-gingival OG). Tooth movement measurements of this Digital Setup (DS) were tabulated for all teeth and used as reference for measuring accuracy of the three different software packages. The DS were exported as STL model files and termed (T2) (Fig. 2).

T1 and T2 models were imported as STL files to the tooth measuring software programs, for registration and 3D linear measurements. The three studied software packages were:

1. Geomagic (G)-(Geomagic U.S., Research Triangle Park, NC) using landmark based method followed by regional global surface closest point registration algorithm (ICP) [17].
2. OrthoAnalyzer (O)-(3Shape Ortho System, Copenhagen, Denmark) using surface 3-point method of registration [18].
3. eModel 9.0 “Compare” (C)-(Geodigm Corporation, Chanhassen, MN) using automatic surface to surface closest point registration algorithm (ICP) [19].

After importing all STL files into the different software packages, the following steps were conducted before measurements were made: 1. Registration, 2. Coordinate system generation, 3. Measurement of tooth movement

1. Registration of the initial model and the Digital Setup using the three software packages

**Fig. 3:**
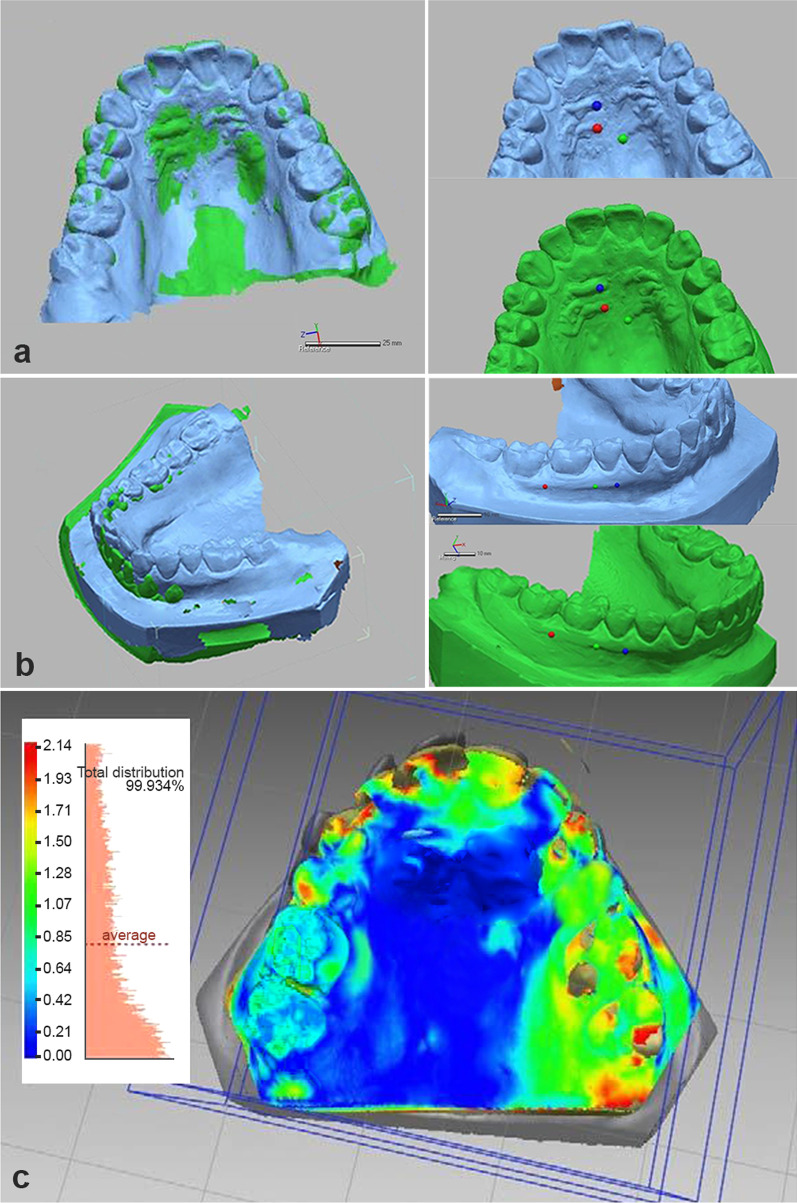
Registration of **a** maxillary models, **b** mandibular models, **c** corresponding heat maps by Geomagic software. Three points on the medial ends of third and second rugae areas in the maxillary arch and three points on the mucogingival junction (MGJ) between first premolar and second premolar, second premolar and first molar, first molar and second molar were selected as reference landmarks, followed by a global and fine regional surface registration

*Geomagic software (G):* [17] Registration was done in two steps (Fig. 3).

1. Landmark based registration: The program was instructed to carry out an initial superimposition based on 3 points on the medial ends of third and second rugae areas in the maxillary arch and 3 points on the mucogingival junction (MGJ) between first premolar and second premolar, second premolar and first molar, first molar and second molar.
2. Global and fine regional surface registration: The program made fine-tuned automatic adjustments to the spatial position of two models based on all points and changes the coordinates of one object to match the other using a best-fit surface algorithm.

**Fig. 4:**
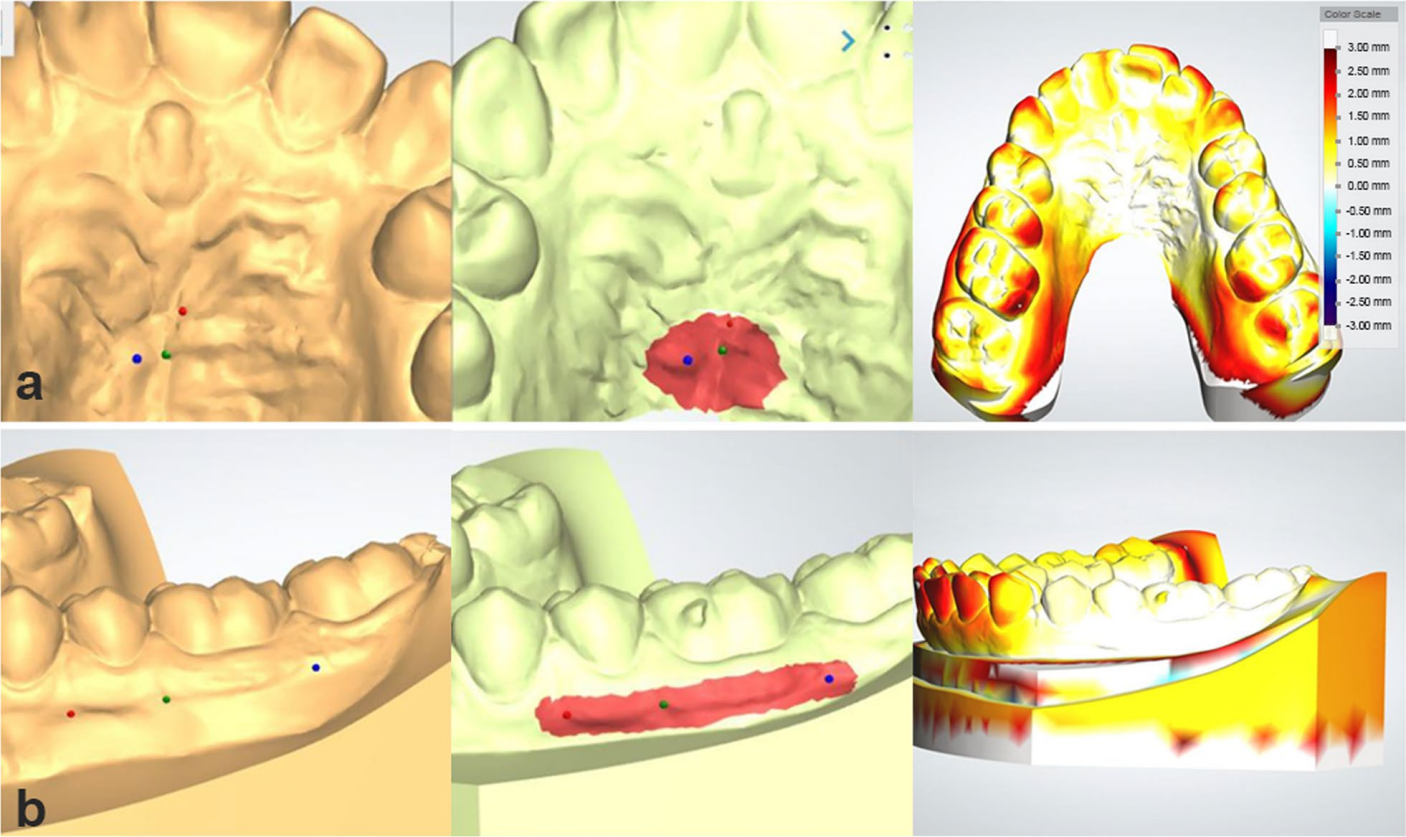
Registration of **a** maxillary models, **b** mandibular models with corresponding heat maps by OrthoAnalyzer software. In the maxillary arch, three points on the medial ends of third and second rugae areas, plus an area of the palate limited anteriorly by the medial 2/3 of the third rugae and laterally by two lines parallel to the mid-palatal suture were used as the landmark. In the mandibular arch, three points on the MGJ between first premolar and second premolar, second premolar and first molar, first molar and second molar, plus an area 1 mm above and below the selected points on the MGJ

*OrthoAnalyzer software (O):* [18] (Fig. 4)

Registration was done using surface 3-point method which involved selecting three landmarks on each of the corresponding models followed by painting an area of known stability to be used for surface-based registration. In the maxillary arch, 3 points on the medial ends of third and second rugae areas, plus an area of the palate limited anteriorly by the medial 2/3 of the third rugae and laterally by two lines parallel to the mid-palatal suture were used as the landmark. In the mandibular arch, 3 points on the MGJ between first premolar and second premolar, second premolar and first molar, first molar and second molar, plus an area 1 mm above and below the selected points on the MGJ.

**Fig. 5:**
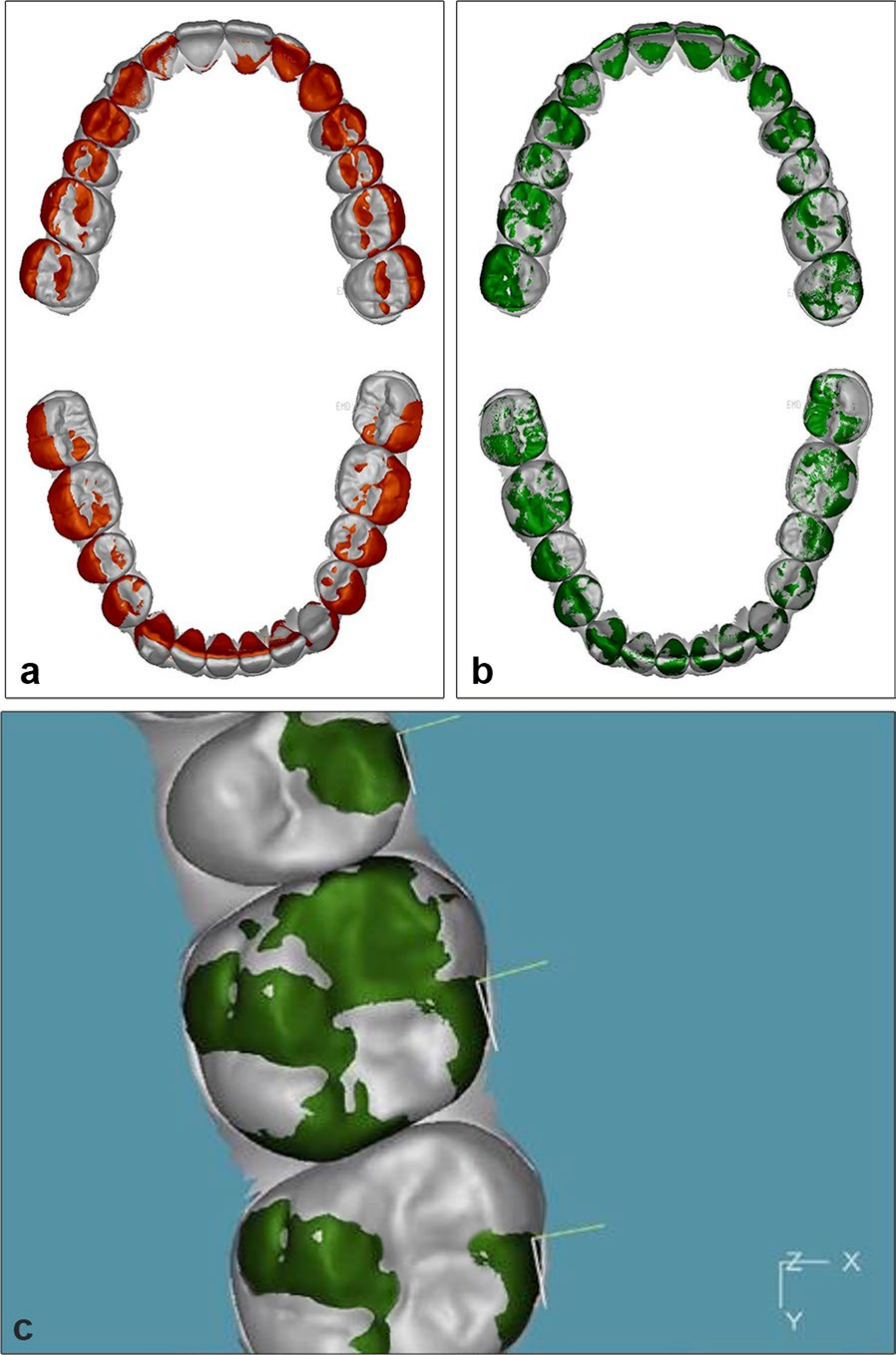
Registration of maxillary models and mandibular models by Compare software. **a** Global alignment of maxillary and mandibular T2 (orange) over T1 model (white). **b** Superimposition of individual segmented teeth from T2 (green) over the unsegmented T1 model (white). **c** Placement of coordinates at the center of resistance of each tooth

*Compare software (C):* [19] Registration was done in three steps (Fig. 5).

1. Model trimming and segmentation of individual teeth: To ensure that the analysis was based solely on tooth-surface features, interproximal papillae and the model base apical to the gingival margin were removed from both the T2 and T1 models. T2 models were then segmented to isolate each tooth as a separate object for superimposition on unsegmented T1 models.
2. Global initial alignment: The dental arches were initially aligned globally, by three-points based on the mesial-buccal cusps of the first molars and the mesial-incisal point of the right central incisor in each arch that were used as matching points for initial registration. Alignment was based on a predefined fit region, which consisted of the occlusal surfaces of the first molars and premolars, tips of canines, and incisal edges of lateral and central incisors. This region was chosen to represent the average occlusal plane for each arch. This initial registration was then refined by 30 iterations of a closest-point algorithm to achieve best fit of the occlusal surfaces.
3. Best fit surface registration: The software then automatically superimposed individual teeth from the segmented T2 models on the corresponding teeth in the unsegmented T1 models using best-fit surface-based registration.

2. Generation of 3D coordinate system and orthogonal planes for tooth movement measurements

After registration, a three-dimensional (3D) coordinate system along the 3 principal axes was generated for tooth movement measurements. According to the software used, either model (Geomagic, OrthoAnalyzer) or tooth (Compare) global reference frames were generated. Model global reference frames are defined as a coordinate system of three mutually perpendicular, intersecting axes (x = sagittal/ anteroposterior, y = vertical/occluso-gingival, and z = transverse/mediolateral). The “x-axis” is defined as the intersection of sagittal and occlusal planes, the “y-axis” as the intersection of the sagittal and coronal planes and the “z-axis” as the intersection of the coronal and occlusal planes [28]. Orthogonal planes defined by the 3 principal axes were also generated.

The 3D planes of space are the transverse plane (occlusal plane) (XZ) which is defined as the plane passing through cusps of bilateral maxillary first molars, second premolars and first premolars. Midsagittal plane (XY) which is defined as the plane perpendicular to the transverse plane and passing through the median palatine raphe, and finally, the frontal plane (coronal) (YZ) which is the plane perpendicular to both the transverse and midsagittal planes. The midsagittal plane was constructed on the mid-palatal suture of the maxilla and then subsequently transferred to the mandible.

For Geomagic, one global model reference frame with the three mutually perpendicular intersecting axes (X, Y, Z) and orthogonal planes was constructed to measure all tooth movements (Composite Model Coordinates). On the other hand, for OrthoAnalyzer, each tooth required the generation of its own spatial model reference frame with the corresponding axes, and planes generated individually to measure tooth movements (Repeated Model Coordinates). However, for Compare, a local tooth reference frame that the software automatically generates, defining the principal local coordinate tooth axes was generated (Automated Tooth Coordinates). The axes were x = mesiodistal (red), y = buccolingual (green), and z = occluso-gingival (blue). Once the axes were placed on the T1 model, the software automatically generated analogous axes for each corresponding tooth in the T2 model.

3. 3D tooth movement measurements

After all digital models (T1 and T2) were oriented in the same coordinate system via registration, it was possible to evaluate how the tooth positions changed.

Measurements made were:

Linear measurements

The change in the translation of each tooth between pretreatment models (T1) and their digital setups (T2) was measured in mm. These were [28]:

a Buccolingual (BL) translation.
b Mesiodistal (MD) translation.
c Occlusogingival (OG) translation.

The measured linear changes were recorded in Excel (Microsoft Excel: 2016 Microsoft Corporation) for comparisons with similar measurements taken from the three studied software packages.

### Intra and inter-examiner reliability

Initially, one researcher (SA) performed all registrations of pretreatment scans with their Digital Setups, reference landmarks and axes identification, modification of local coordinates, as well as all tooth movement measurements. The same and another calibrated investigator (NV) repeated the measurements on 5 randomly selected scan sets 4 weeks later to test reliability. All measures were pooled to give a summary estimate to calculate Intraclass Correlation Coefficients for intra-examiner and inter-examiner reliability.

### Statistical analysis of the data

Statistical analysis was carried out using IBM SPSS software package version 20.0. (Armonk, NY: IBM Corp). Data from individual teeth were pooled to provide an overall estimate of the amount of tooth movement in each degree of freedom and summarized as mean and standard deviation. Two-way fixed-rater single-measure Intraclass Correlation Coefficients (ICC) of absolute agreement were calculated between the pooled amount of tooth movement in each degree of freedom measured by each software package and the amount of tooth movement from the digital setup (reference standard). Overall agreement between the three software packages were similarly calculated. Based on the 95% confident interval of the ICC estimate, values less than 0.5, between 0.5 and 0.75, between 0.75 and 0.9, and greater than 0.90 are indicative of poor, moderate, good, and excellent reliability, respectively based on “A Guideline of Selecting and Reporting Intraclass Correlation Coefficients for Reliability Research” by Koo and Li [29]. Statistical significance of the obtained results was expressed at p ≤ 0.01 to account for multiple comparisons.

## Results

Intra-examiner reliability for Geomagic, OrthoAnalyzer and Compare software were 0.941, 0.899, and 0.978 respectively. As for inter-examiner reliability, they were 0.926, 0.798, and 0.944 respectively.

Table 1 shows the descriptive statistics (mean and standard deviation) for the maxillary and mandibular teeth with respect to the three linear movements for the DS and the three tested software (Geomagic, OrthoAnalyzer and Compare). Agreement between each package and the reference standard are presented as ICC in Table 2.

**Table 1:** Amount of linear tooth movement measured in mm as determined by each software package

Type	Movements	No	DS Mean ± S.D	Geomagic Mean ± S.D	OrthoAnalyzer Mean ± S.D	Compare Mean ± S.D
Maxillary	OG	166	0.508 ± 0.8	0.470 ± 0.8	0.244 ± 0.5	0.397 ± 0.7
	MD	225	− 0.203 ± 1.0	− 0.177 ± 0.9	− 0.045 ± 0.6	− 0.132 ± 0.8
	BL	212	− 0.138 ± 1.4	− 0.133 ± 1.3	− 0.076 ± 1.1	− 0.115 ± 1.2
Mandibular	OG	176	0.861 ± 0.9	0.767 ± 0.8	0.641 ± 0.8	0.809 ± 0.8
	MD	216	− 0.209 ± 1.1	− 0.186 ± 1.0	− 0.162 ± 0.7	− 0.196 ± 1.0
	BL	190	− 0.129 ± 1.3	− 0.097 ± 1.2	− 0.058 ± 1	− 0.108 ± 1.2
Overall	OG	342	0.690 ± 0.9	0.623 ± 0.8	0.448 ± 0.7	0.609 ± 0.8
	MD	441	− 0.206 ± 1.0	− 0.181 ± 0.9	− 0.102 ± 0.7	− 0.163 ± 0.9
	BL	402	− 0.134 ± 1.3	− 0.116 ± 1.2	− 0.067 ± 1.1	− 0.112 ± 1.2

Data was expressed using Mean ± SD

DS Digital Setup, OG Occlusogingival, MD Mesiodistal, BL Buccolingual

**Table 2:** Intraclass Correlation Coefficients for different movements among the three software packages, in comparison to the digital setup

Movements	Type	Digital Setup vs. Geomagic		Digital Setup vs. OrthoAnalyzer		Digital Setup vs. Compare	
		ICC	95% CI	ICC	95% CI	ICC	95% CI
OG	Maxillary	0.958^*^	0.944–0.969	0.663^*^	0.452–0.784	0.901^*^	0.851–0.932
	Mandibular	0.869^*^	0.825–0.902	0.552^*^	0.431–0.652	0.910^*^	0.881–0.933
	Overall	0.911^*^	0.889–0.928	0.615^*^	0.501–0.701	0.911^*^	0.887–0.930
MD	Maxillary	0.965^*^	0.955–0.973	0.670^*^	0.582–0.740	0.905^*^	0.877–0.927
	Mandibular	0.871^*^	0.834–0.900	0.589^*^	0.494–0.669	0.917^*^	0.893–0.936
	Overall	0.914^*^	0.897–0.928	0.624^*^	0.563–0.679	0.912^*^	0.894–0.926
BL	Maxillary	0.968^*^	0.959–0.976	0.730^*^	0.660–0.787	0.920^*^	0.896–0.938
	Mandibular	0.873^*^	0.834–0.903	0.606^*^	0.508–0.689	0.925^*^	0.901–0.943
	Overall	0.928^*^	0.913–0.940	0.676^*^	0.619–0.726	0.922^*^	0.906–0.935

ICC Intraclass Correlation Coefficient, CI Confidence Interval, LL: Lower Limit, UL Upper Limit, OG Occlusogingival, MD Mesiodistal, BL Buccolingual

^*^All values were significant at p ≤ 0.001∙

BL linear movement showed the highest agreements in all three software packages (Overall ICC **G:** 0.928, **O:** 0.676, **C:** 0.922), followed by comparable values for agreements for the MD (Overall ICC **G:** 0.914, **O:** 0.624, **C:** 0.912) and OG movements (Overall ICC **G:** 0.911, **O:** 0.615, **C:** 0.911).

For Geomagic software, the maxillary ICC for all linear measurements showed excellent agreements with the DS (> 0.90), while the mandibular ICCs for all measurements showed good agreements (0.75–0.90). In OrthoAnalyzer software, all maxillary and mandibular linear ICCs showed only moderate values for agreements (0.50–< 0.75). As for Compare software, all maxillary and mandibular linear measurements showed excellent agreements with the DS (> 0.90).

## Discussion

Quantifying the amount of orthodontic tooth movement in digital orthodontics is performed by registration software packages, which are dependent on the registration algorithm used [10, 30]. Scholarly literature lacks studies testing accuracy of software packages to compare treatment effects and efficacy [5, 8, 10, 19–21, 31–35]. The present study calculated the amount of tooth movement in the three linear directions for the entire maxillary and mandibular dentitions including second permanent molars.

Since tooth movements on the Digital Setup were performed by the principal investigator, the true value for translation *(type, direction and amount)* for each tooth could be used as a reference [13, 20, 36]. The reliability and validity of using Digital Setup generated by OrthoAnalyzer software was previously studied and it was concluded that it is as effective and accurate as manual setups and represents an efficient tool for diagnosis and appliance fabrication, that can be reliably reproduced [37, 38]. The present study used reference landmarks and area on the rugae for registration of the maxillary digital models in two of the three software packages (Geomagic, OrthoAnalyzer) that required reference structures. The selected landmarks have been documented previously in several studies to be considered as stable landmarks for maxillary digital model registration [11, 22, 23, 39]. In the mandibular arch, landmarks on the MGJ were used both in Geomagic and OrthoAnalyzer softwares. This was based on the findings by Ioshida et al. [40], who concluded that MGJ can be used as a reference area of good stability for mandibular superimpositions. However, the Compare software did not require the identification of a reference point or area for either arch outside the dentition.

The Compare software showed higher mandibular Intraclass Correlation Coefficients (ICCs) for all movements in comparison to their maxillary equivalents. In addition, mandibular ICCs were always greater for Compare software than the other two software. The Compare software does not require anatomical landmark or surface selection before registration but rather depends on the automatic superimposition tool of the software after initial global alignment. Mandibular ICCs were consistently lower than their maxillary equivalents for all linear movements in the two software (Geomagic and Ortho Analyzer) that required either landmark or surface selection for the registration. While Compare removes the interproximal papillae and model base apical to the gingival margin to ensure that the registration is based solely on tooth-surface features. This suggests that the mandibular superimposition using the MGJ landmarks with an area around it as a reference is less accurate than the maxillary superimposition using the rugae area. Only two studies have attempted to study stable landmarks and areas to be used for accurate and reliable mandibular superimposition [24, 40]. On the other hand, numerous studies have endorsed the accuracy of maxillary reference points and areas to be used for maxillary digital superimposition [11–13, 21, 22, 26, 39, 41–43].

The current study employed registration techniques as mandated by the algorithms employed by the various software packages [16]. The registration technique for the Geomagic and Compare software used a best-fit method [20, 22, 31, 42]. This technique of ‘fine matching’ uses thousands of reference points instead of a few landmarks/area and is based on ‘Iterative Closest Point Algorithms’ (ICP) [44]. The effect of outliers is decreased while accuracy markedly improves. Although OrthoAnalyzer uses a surface-based registration technique, it doesn’t use an algorithm that iterates to improve the overall quality of registration, unlike the ICP employed in the Geomagic and Compare software. This could explain the higher ICC values for Geomagic and Compare software compared to OrthoAnalyzer software.

The lowest ICC values for all measured linear movements with OrthoAnalyzer software could be attributed to a crucial factor for digital superimpositions which is to generate an accurate and reproducible coordinate system. Geomagic software requires only one global coordinate system, Compare software generates automated computations for placement of local coordinate systems at each tooth’s approximate center of resistance [10, 19, 33], while OrthoAnalyzer software required creation of multiple customized global coordinates for each tooth. An assumption can be made that the coordinate system employed by OrthoAnalyzer will be more accurate because it is customized for each tooth according to its location in the dental arch. However, this assumption was not true as it induced more operator error each time a plane was constructed for measurements.

### Linear measurements

In the present study, Geomagic and OrthoAnalyzer software packages used surface landmarks, since it was not possible to place a centroid on T1 and T2 and measure the differential positions using these software packages. While Compare software used automatic derived coordinates at the centroid. Current literature describes tooth movement in digital superimpositions based on the movement of different landmarks on a tooth. Some authors used cusp tips and incisal edges [12, 42], like what we used in the present study. Although in theory it is reliable to locate a landmark on a cusp tip, its displacement represents only the displacement of that landmark and not the displacement of the whole tooth, therefore, not differentiating between tipping and bodily movement [23, 31, 42]. Studies with landmarks averaged to a centroid were able to describe the translational movements of teeth but not report rotational changes [41, 45]. Additionally, Sandler used a shell to calculate molar movement keeping the buccal and palatal outlines similar between T1 and T2, to get rid of any gingival effects on the posttreatment model (e.g., gingival irritation, overgrowth, or hyperplasia), which would affect the determination of the center of mass (Co M) of the crown [32]. The difference in landmark location between the studied software packages could clarify the excellent agreement for all linear movements measured by Compare software in both arches in comparison to the other software packages. Though both of Geomagic and OrthoAnalyzer software packages used surface landmarks, the ICC values of Geomagic demonstrated far better agreements. This could be attributed to the fact that in Geomagic, a true 3D distance was measured between T1 and T2 landmarks compared to a constructed plane in OrthoAnalyzer. Additionally, the brush size that was used to locate landmarks in Geomagic  software is much smaller in size than the one used in OrthoAnalyzer software, rendering point location with greater precision.

Buccolingual movements showed slightly higher agreements in the three tested software packages in comparison to other linear measurements, with fairly close ICC values between the MD and OG movements. This was in agreement with Choi et al. [42], who reported that the three linear measurements did not differ significantly upon comparing plaster and digital models.

Based on the findings of the current study, Compare software package showed the greatest advantage in terms of accuracy compared to the other software packages.

## Conclusions

1. Compare and Geomagic software packages consistently showed maximum accuracy in measuring the amount of tooth movement in the maxillary arch compared to the reference standard.
2. Compare software showed highest agreements in the mandibular arch.
3. None of the studied software packages showed poor agreement with the DS across all tooth movement measurements in both arches.
4. Buccolingual displacement was the movement recorded with highest accuracy and agreement between software packages.

### Limitations of the present study

1. All measurements were based on the morphology of the clinical crown due to the absence of roots in intraoral scans, therefore the tooth centroid could not be defined. The linear measurements calculated cannot determine the type of tooth movement whether translation, or rotation or a combination of both.
2. Using the digital setup as a reference standard maximize the chance of agreement with the registration software packages since the adjacent soft tissues are not altered. Accounting for the tissue changes concomitant with orthodontic tooth movement, the accuracy of the registration software packages will likely be lower than the reported values.

## Data Availability

All data generated or analysed during this study are included in this published article in the form of tables and figures.

## References

[1] Hayashi K, Uechi J, Lee S-P, Mizoguchi I (2007). Three-dimensional analysis of orthodontic tooth movement based on XYZ and finite helical axis systems. Eur J Orthod.

[2] Gkantidis N, Schauseil M, Pazera P, Zorkun B, Katsaros C, Ludwig B (2015). Evaluation of 3-dimensional superimposition techniques on various skeletal structures of the head using surface models. PLoS ONE.

[3] Stucki S, Gkantidis N (2020). Assessment of techniques used for superimposition of maxillary and mandibular 3D surface models to evaluate tooth movement: a systematic review. Eur J Orthod.

[4] Vaid NR (2018). Digital technologies in orthodontics 2013; an update. Semin Orthod.

[5] Haouili N, Kravitz ND, Vaid NR, Ferguson DJ, Makki L (2020). Has Invisalign improved? A prospective follow-up study on the efficacy of tooth movement with Invisalign. Am J Orthod Dentofacial Orthop.

[6] Cha BK, Choi JI, Jost-Brinkmann PG, Jeong YM (2007). Applications of three-dimensionally scanned models in orthodontics. Int J Comput Dent.

[7] De Luca CG, Pachêco-Pereira C, Lagravere MO, Flores-Mir C, Major PW (2015). Intra-arch dimensional measurement validity of laser-scanned digital dental models compared with the original plaster models: a systematic review. Orthod Craniofac Res.

[8] Camardella LT, Ongkosuwito EM, Penning EW, Kuijpers-Jagtman AM, Vilella OV, Breuning KH (2020). Accuracy and reliability of measurements performed using two different software programs on digital models generated using laser and computed tomography plaster model scanners. Korean J Orthod.

[9] Araújo TM, Fonseca LM, Caldas LD, Costa-Pinto RA (2012). Preparation and evaluation of orthodontic setup. Dental Press J Orthod.

[10] Grauer D, Proffit WR (2011). Accuracy in tooth positioning with a fully customized lingual orthodontic appliance. Am J Orthod Dentofacial Orthop.

[11] Vasilakos G, Schilling R, Halazonetis D (2017). Assessment of different techniques for 3D superimposition of serial digital maxillary dental casts on palatal structures. Sci Rep.

[12] Talaat S, Kaboudan A, Bourauel C, Ragy N, Kula K, Ghoneima A (2017). Validity and reliability of three-dimensional palatal superimposition of digital dental models. Eur J Orthod.

[13] Ganzer N, Feldmann I, Liv P, Bondemark L (2018). A novel method for superimposition and measurements on maxillary digital 3D models-studies on validity and reliability. Eur J Orthod.

[14] Bichu YM, Hansa I, Bichu AY, Premjani P, Flores-Mir C, Vaid NR (2021). Applications of artificial intelligence and machine learning in orthodontics: a scoping review. Prog Orthod.

[15] Vaid NR (2021). Artificial Intelligence (AI) driven orthodontic care: a quest toward utopia?. Semin Orthod.

[16] Oliveira FP, Tavares JM (2014). Medical image registration: a review. Comput Methods Biomech Biomed Engin.

[17] Geomagic. Geomagic design X user guide (2013). https://www.engineering.pitt.edu/uploadedFiles/_Content/Sub_Sites/Business/MRW/SCPI/_Library/specs/geomagicdesignx2014userguide.pdf.

[18] 3 Shape Ortho System. OrthoAnalyzer 2012 User Manual (2012). http://promed.ua/wp-content/uploads/2012/01/2012_OrthoAnalyzer_English.pdf.

[19] Awad MG, Ellouze S, Ashley S, Vaid N, Makki L, Ferguson DJ (2018). Accuracy of digital predictions with CAD/CAM labial and lingual appliances: a retrospective cohort study. Semin Orthod.

[20] Thiruvenkatachari B, Al-Abdallah M, Akram NC, Sandler J, O'Brien K (2009). Measuring 3-dimensional tooth movement with a 3-dimensional surface laser scanner. Am J Orthod Dentofacial Orthop.

[21] Choi JI, Cha BK, Jost-Brinkmann PG, Choi DS, Jang IS (2012). Validity of palatal superimposition of 3-dimensional digital models in cases treated with rapid maxillary expansion and maxillary protraction headgear. Korean J Orthod.

[22] Chen G, Chen S, Zhang XY, Jiang RP, Liu Y, Shi FH (2011). Stable region for maxillary dental cast superimposition in adults, studied with the aid of stable miniscrews. Orthod Craniofac Res.

[23] Jang I, Tanaka M, Koga Y, Iijima S, Yozgatian JH, Cha BK (2009). A novel method for the assessment of three-dimensional tooth movement during orthodontic treatment. Angle Orthod.

[24] An K, Jang I, Choi DS, Jost-Brinkmann PG, Cha BK (2015). Identification of a stable reference area for superimposing mandibular digital models. J Orofac Orthop.

[25] Kottner J, Audigé L, Brorson S, Donner A, Gajewski BJ, Hróbjartsson A (2011). Guidelines for Reporting Reliability and Agreement Studies (GRRAS) were proposed. J Clin Epidemiol.

[26] Talaat S, Kaboudan A, Breuning H, Ragy N, Elshebiny T, Kula K (2015). Reliability of linear and angular dental measurements with the OrthoMechanics Sequential Analyzer. Am J Orthod Dentofacial Orthop.

[27] Walter S, Eliasziw M, Donner A (1998). Sample size and optimal designs for reliability studies. Stat Med.

[28] Daskalogiannakis J. Glossary of orthodontic terms Chicago: Quintessence Pub. Co. (2000). http://books.google.com/books?id=DOtpAAAAMAAJ.

[29] Koo TK, Li MY (2016). A guideline of selecting and reporting intraclass correlation coefficients for reliability research. J Chiropr Med.

[30] Gandedkar NH, Vaid NR, Darendeliler MA, Premjani P, Ferguson DJ (2019). The last decade in orthodontics: a scoping review of the hits, misses and the near misses!. Semin Orthod.

[31] Cha BK, Lee JY, Jost-Brinkmann PG, Yoshida N (2007). Analysis of tooth movement in extraction cases using three-dimensional reverse engineering technology. Eur J Orthod.

[32] Sandler J, Thiruvenkatachari B, Gutierrez R (2017). Measuring molar movement: a reliable technique. APOS Trends Orthod.

[33] Grünheid T, Loh C, Larson BE (2017). How accurate is invisalign in nonextraction cases? Are predicted tooth positions achieved?. Angle Orthod.

[34] Sachdev S, Tantidhnazet S, Saengfai NN (2021). Accuracy of tooth movement with in-house clear aligners. J World Fed Orthod.

[35] Chong DR, Jang YJ, Chun YS, Jung SH, Lee SK (2005). The evaluation of rotational movements of maxillary posterior teeth using three dimensional images in cases of extraction of maxillary first premolar. Korean J Orthod.

[36] Chen J, Li S, Fang S (2009). Quantification of tooth displacement from cone-beam computed tomography images. Am J Orthod Dentofacial Orthop.

[37] Camardella LT, Rothier EK, Vilella OV, Ongkosuwito EM, Breuning KH (2016). Virtual setup: application in orthodontic practice. J Orofac Orthop.

[38] Barreto MS, Faber J, Vogel CJ, Araujo TM (2016). Reliability of digital orthodontic setups. Angle Orthod.

[39] Shukla D, Chowdhry A, Bablani D, Jain P, Thapar R (2011). Establishing the reliability of palatal rugae pattern in individual identification (following orthodontic treatment). J Forensic Odontostomatol.

[40] Ioshida M, Muñoz BA, Rios H, Cevidanes L, Aristizabal JF, Rey D (2019). Accuracy and reliability of mandibular digital model registration with use of the mucogingival junction as the reference. Oral Surg Oral Med Oral Pathol Oral Radiol Endod.

[41] Ashmore JL, Kurland BF, King GJ, Wheeler TT, Ghafari J, Ramsay DS (2002). A 3-dimensional analysis of molar movement during headgear treatment. Am J Orthod Dentofacial Orthop.

[42] Choi DS, Jeong YM, Jang I, Jost-Brinkmann PG, Cha BK (2010). Accuracy and reliability of palatal superimposition of three-dimensional digital models. Angle Orthod.

[43] Garib D, Miranda F, Yatabe MS, Lauris JRP, Massaro C, McNamara JA (2019). Superimposition of maxillary digital models using the palatal rugae: does ageing affect the reliability?. Orthod Craniofac Res.

[44] Pomerleau F, Colas F, Siegwart R, Magnenat S (2013). Comparing ICP variants on real-world data sets. Auton Robots.

[45] Lai EH, Yao CC, Chang JZ, Chen I, Chen YJ (2008). Three-dimensional dental model analysis of treatment outcomes for protrusive maxillary dentition: comparison of headgear, miniscrew, and miniplate skeletal anchorage. Am J Orthod Dentofacial Orthop.

